# Supplementary material for: HIVprotI: an integrated web based platform for prediction and design of HIV proteins inhibitors
Source: J Cheminform. 2018 Mar 9;10:12. doi: 10.1186/s13321-018-0266-y (PMC5845081; doi:10.1186/s13321-018-0266-y)
Supplement: Supplementary file 2 — Additional file 2. Source code of HIVProtI web server. [file 13321_2018_266_MOESM2_ESM.zip › HIVprotI_Source-code/hivprotI/weka-3-6-6/doc/weka/gui/graphvisualizer/HierarchicalBCEngine.html]

HierarchicalBCEngine


---


|  |  |  |
| --- | --- | --- |
| **Overview** \| **Package** \| **Class** \| **Tree** \| **Deprecated** \| **Index** \| **Help** \| **Weka's home** | |  |
| **PREV CLASS**   **NEXT CLASS** | **FRAMES**    **NO FRAMES**     **All Classes** |  |
| SUMMARY: NESTED \| FIELD \| CONSTR \| METHOD | DETAIL: FIELD \| CONSTR \| METHOD |  |


---


## weka.gui.graphvisualizer Class HierarchicalBCEngine

```
java.lang.Object
  weka.gui.graphvisualizer.HierarchicalBCEngine
```

**All Implemented Interfaces:**: GraphConstants, LayoutEngine

---

``` public class HierarchicalBCEngine extends java.lang.Object implements GraphConstants, LayoutEngine ```

This class lays out the vertices of a graph in a
hierarchy of vertical levels, with a number of nodes
in each level. The number of levels is the depth of
the deepest child reachable from some parent at level
0. It implements a layout technique as described by
K. Sugiyama, S. Tagawa, and M. Toda. in "Methods for
visual understanding of hierarchical systems", IEEE
Transactions on Systems, Man and Cybernetics,
SMC-11(2):109-125, Feb. 1981.

There have been a few modifications made, however.
The crossings function is changed as it was non-linear
in time complexity. Furthermore, we don't have any
interconnection matrices for each level, instead we
just have one big interconnection matrix for the
whole graph and a int[][] array which stores the
vertices present in each level.

**Version:**
:   $Revision: 7059 $ - 24 Apr 2003 - Initial version (Ashraf M. Kibriya)

**Author:**
:   Ashraf M. Kibriya (amk14@cs.waikato.ac.nz)

---

**Field Summary**

**Fields inherited from interface weka.gui.graphvisualizer.GraphConstants**

`DIRECTED, DOUBLE, NORMAL, PLURAL_DUMMY, REVERSED, SINGULAR_DUMMY`


**Constructor Summary**

`HierarchicalBCEngine()`
  
          SimpleConstructor
If we want to instantiate the class first, and if information for
nodes and edges is not available.

`HierarchicalBCEngine(FastVector nodes,
FastVector edges,
int nodeWidth,
int nodeHeight)`
  
          Constructor - takes in FastVectors of nodes and edges, and the initial
width and height of a node

`HierarchicalBCEngine(FastVector nodes,
FastVector edges,
int nodeWidth,
int nodeHeight,
boolean edgeConcentration)`
  
          Constructor - takes in FastVectors of nodes and edges, the initial width
and height of a node, and a boolean value to indicate if the edges
should be concentrated.


| **Method Summary** | |
| --- | --- |
| `void` | `addLayoutCompleteEventListener(LayoutCompleteEventListener l)`             Method to add a LayoutCompleteEventListener |
| `void` | `fireLayoutCompleteEvent(LayoutCompleteEvent e)`             Fires a LayoutCompleteEvent. |
| `javax.swing.JPanel` | `getControlPanel()`             This method returns a handle to the extra controls panel, so that the visualizing class can add it to some of it's own gui panel. |
| `FastVector` | `getNodes()`             give access to set of graph nodes |
| `javax.swing.JProgressBar` | `getProgressBar()`             Returns a handle to the progressBar of this LayoutEngine. |
| `void` | `layoutGraph()`             This method does a complete layout of the graph which includes removing cycles, assigning levels to nodes, reducing edge crossings and laying out the vertices horizontally for better visibility. |
| `void` | `phaseIID(int lindex, int[][] levels)`             See Sugiyama et al. |
| `void` | `phaseIIU(int lindex, int[][] levels)`             See Sugiyama et al. |
| `void` | `phaseIU(int lindex, int[][] levels)`             See Sugiyama et al. |
| `void` | `removeLayoutCompleteEventListener(LayoutCompleteEventListener e)`             Method to remove a LayoutCompleteEventListener. |
| `void` | `setNodesEdges(FastVector nodes, FastVector edges)`             Sets the nodes and edges for this LayoutEngine. |
| `void` | `setNodeSize(int nodeWidth, int nodeHeight)`             Sets the size of a node. |

**Methods inherited from class java.lang.Object**

`equals, getClass, hashCode, notify, notifyAll, toString, wait, wait, wait`

**Constructor Detail**

### HierarchicalBCEngine

```
public HierarchicalBCEngine(FastVector nodes,
                            FastVector edges,
                            int nodeWidth,
                            int nodeHeight)
```

:   Constructor - takes in FastVectors of nodes and edges, and the initial
    width and height of a node

---


### HierarchicalBCEngine

```
public HierarchicalBCEngine(FastVector nodes,
                            FastVector edges,
                            int nodeWidth,
                            int nodeHeight,
                            boolean edgeConcentration)
```

:   Constructor - takes in FastVectors of nodes and edges, the initial width
    and height of a node, and a boolean value to indicate if the edges
    should be concentrated.

    **Parameters:**: `nodes` - - FastVector containing all the nodes: `edges` - - FastVector containing all the edges: `nodeWidth` - - A node's allowed width: `nodeHeight` - - A node's allowed height: `edgeConcentration` - - True: if want to concentrate edges, False: otherwise

---


### HierarchicalBCEngine

```
public HierarchicalBCEngine()
```

:   SimpleConstructor
    If we want to instantiate the class first, and if information for
    nodes and edges is not available. However, we would have to manually
    provide all the information later on by calling setNodesEdges and
    setNodeSize methods


**Method Detail**

### getNodes

```
public FastVector getNodes()
```

:   give access to set of graph nodes

    :   **Specified by:**: `getNodes` in interface `LayoutEngine`

---


### getControlPanel

```
public javax.swing.JPanel getControlPanel()
```

:   This method returns a handle to the extra
    controls panel, so that the visualizing
    class can add it to some of it's own
    gui panel.

    :   **Specified by:**: `getControlPanel` in interface `LayoutEngine`

---


### getProgressBar

```
public javax.swing.JProgressBar getProgressBar()
```

:   Returns a handle to the progressBar
    of this LayoutEngine.

    :   **Specified by:**: `getProgressBar` in interface `LayoutEngine`

---


### setNodesEdges

```
public void setNodesEdges(FastVector nodes,
                          FastVector edges)
```

:   Sets the nodes and edges for this LayoutEngine.
    Must be used if the class created by simple
    HierarchicalBCEngine() constructor.

    :   **Specified by:**: `setNodesEdges` in interface `LayoutEngine`
    :   **Parameters:**: `nodes` - - FastVector containing all the nodes: `edges` - - FastVector containing all the edges

---


### setNodeSize

```
public void setNodeSize(int nodeWidth,
                        int nodeHeight)
```

:   Sets the size of a node. This method must be
    used if the class created by simple
    HierarchicalBCEngine() constructor.

    :   **Specified by:**: `setNodeSize` in interface `LayoutEngine`
    :   **Parameters:**: `nodeWidth` - - A node's allowed width: `nodeHeight` - - A node's allowed height

---


### addLayoutCompleteEventListener

```
public void addLayoutCompleteEventListener(LayoutCompleteEventListener l)
```

:   Method to add a LayoutCompleteEventListener

    :   **Specified by:**: `addLayoutCompleteEventListener` in interface `LayoutEngine`
    :   **Parameters:**: `l` - - Listener to receive the LayoutCompleteEvent by this class.

---


### removeLayoutCompleteEventListener

```
public void removeLayoutCompleteEventListener(LayoutCompleteEventListener e)
```

:   Method to remove a LayoutCompleteEventListener.

    :   **Specified by:**: `removeLayoutCompleteEventListener` in interface `LayoutEngine`
    :   **Parameters:**: `e` - - The LayoutCompleteEventListener to remove.

---


### fireLayoutCompleteEvent

```
public void fireLayoutCompleteEvent(LayoutCompleteEvent e)
```

:   Fires a LayoutCompleteEvent.

    :   **Specified by:**: `fireLayoutCompleteEvent` in interface `LayoutEngine`
    :   **Parameters:**: `e` - - The LayoutCompleteEvent to fire

---


### layoutGraph

```
public void layoutGraph()
```

:   This method does a complete layout of the graph which includes
    removing cycles, assigning levels to nodes, reducing edge crossings
    and laying out the vertices horizontally for better visibility.
    The removing of cycles and assignment of levels is only performed
    if hasn't been performed earlier or if some layout option has been
    changed and it is necessary to do so. It is necessary to do so,
    if the user selects/deselects edge concentration or topdown/bottomup
    options.

    The layout is performed in a separate thread and the progress
    bar of the class is updated for each of the steps as the process
    continues.

    :   **Specified by:**: `layoutGraph` in interface `LayoutEngine`

---


### phaseIU

```
public void phaseIU(int lindex,
                    int[][] levels)
```

:   See Sugiyama et al. 1981 (full reference give at top)
    lindex is the index of the level we want to process.
    In this method we'll sort the vertices at the level
    lindex according to their DOWN-barycenters (or row
    barycenters).

---


### phaseIID

```
public void phaseIID(int lindex,
                     int[][] levels)
```

:   See Sugiyama et al. 1981 (full reference give at top)

---


### phaseIIU

```
public void phaseIIU(int lindex,
                     int[][] levels)
```

:   See Sugiyama et al. 1981 (full reference give at top)


---


|  |  |  |
| --- | --- | --- |
| **Overview** \| **Package** \| **Class** \| **Tree** \| **Deprecated** \| **Index** \| **Help** \| **Weka's home** | |  |
| **PREV CLASS**   **NEXT CLASS** | **FRAMES**    **NO FRAMES**     **All Classes** |  |
| SUMMARY: NESTED \| FIELD \| CONSTR \| METHOD | DETAIL: FIELD \| CONSTR \| METHOD |  |


---
